# Controlling Fragmentation of the Acetylene Cation in the Vacuum Ultraviolet via Transient Molecular Alignment

**DOI:** 10.1021/acs.jpclett.2c03354

**Published:** 2022-12-23

**Authors:** L. Varvarezos, J. Delgado-Guerrero, M. Di Fraia, T. J. Kelly, A. Palacios, C. Callegari, A. L. Cavalieri, R. Coffee, M. Danailov, P. Decleva, A. Demidovich, L. DiMauro, S. Düsterer, L. Giannessi, W. Helml, M. Ilchen, R. Kienberger, T. Mazza, M. Meyer, R. Moshammer, C. Pedersini, O. Plekan, K. C. Prince, A. Simoncig, A. Schletter, K. Ueda, M. Wurzer, M. Zangrando, F. Martín, J. T. Costello

**Affiliations:** † School of Physical Sciences and National Centre for Plasma Science and Technology, Dublin City University, Dublin 9, Ireland; ‡ Departamento de Química, Módulo 13, Universidad Autónoma de Madrid, 28049 Madrid, Spain; § Instituto Madrileño de Estudios Advanzados en Nanociencia, Cantoblanco, 28049 Madrid, Spain; ∥ Elettra-Sincrotrone Trieste S.C.p.A., Basovizza, 34149 Trieste, Italy; ⊥ Department of Computer Science and Applied Physics, Atlantic Technological University, T91 T8NW Galway, Ireland; # Institute for Advanced Research in Chimical Sciences, Universidad Autónoma de Madrid, 28049 Madrid, Spain; ∇ Institute of Applied Physics, University of Bern, 3012 Bern, Switzerland; ○ Paul Scherrer Institute, 5232 Villigen PSI, Switzerland; ◆ Linac Coherent Light Source/SLAC National Accelerator Laboratory, Menlo Park, California 94025, United States; ¶ Istituto Officina dei Materiali IOM-CNR and Dipartimento di Scienze Chimiche e Farmaceutiche, Università degli Studi di Trieste, 34121 Trieste, Italy; ■ Department of Physics, The Ohio State University, Columbus, Ohio 43210, United States; ☆ Deutsches Elektronen-Synchrotron (DESY), Notkestrasse 85, 22607 Hamburg, Germany; ● Fakultät Physik, Technische Universität Dortmund, Maria-Goeppert-Mayer-Str. 2, 44227 Dortmund, Germany; △ Institut fur Physik und CINSaT, Universitat Kassel, Heinrich-Plett-Str. 40, 34132 Kassel, Germany; ★ European XFEL, Holzkoppel 4, 22869 Schenefeld, Germany; ▼ Physics Department, Technische Universität München, 85748 Garching, Germany; ⬡ Max-Planck Institut für Kernphysik, Saupfercheckweg 1, 69117 Heidelberg, Germany; ⊙ Department of Chemistry and Biotechnology, Swinburne University of Technology, Melbourne, Victoria 3122, Australia; ⬢ Institute of Multidisciplinary Research for Advanced Materials, Tohoku University, Sendai 980-8577, Japan; ◇ Istituto Officina dei Materiali, Consiglio Nazionale delle Ricerche, 34149 Trieste, Italy; □ Condensed Matter Physics Center, Universidad Autónoma de Madrid, 28049 Madrid, Spain

## Abstract

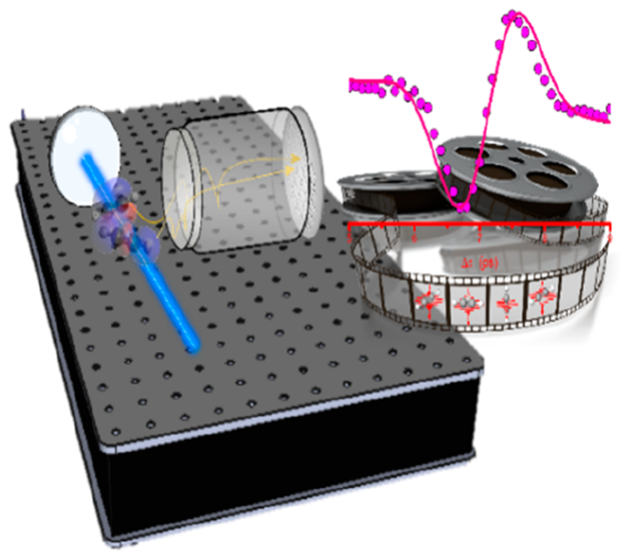

An open-loop control scheme of molecular fragmentation based on transient molecular alignment combined with single-photon ionization induced by a short-wavelength free electron laser (FEL) is demonstrated for the acetylene cation. Photoelectron spectra are recorded, complementing the ion yield measurements, to demonstrate that such control is the consequence of changes in the electronic response with molecular orientation relative to the ionizing field. We show that stable C_2_H_2_^+^ cations are mainly produced when the molecules are parallel or nearly parallel to the FEL polarization, while the hydrogen fragmentation channel (C_2_H_2_^+^ → C_2_H^+^ + H) predominates when the molecule is perpendicular to that direction, thus allowing one to distinguish between the two photochemical processes. The experimental findings are supported by state-of-the art theoretical calculations.

Tracking the evolution of light-induced chemical reactions, including but not limited to molecular fragmentation, polymerization, and photoisomerization, on their natural time scales remains an intriguing quest for femtochemistry. Progress in modern technologies such as molecular photovoltaics,^1^ the development of light-activated CO-release molecules (photo-CORMS),^2,3^ and the production of integrated circuits by means of photolithography^4,5^ critically depend on a thorough understanding of the underlying photochemical processes.

Apart from monitoring photochemical reaction pathways, emphasis has also been placed on exerting active control on them. This can be achieved by using laser field parameters such as the phase and frequency.^6^ In the former case, the so-called Brumer–Shapiro scheme^7^ exploits interferences between different reaction pathways, whereas in the latter case linear variation of the laser frequency as a function of time (chirp) is applied to achieve control of electronic excitations in molecules. In this respect, several open-loop schemes using spectrally and/or temporally tailored ultrashort pulses have been reported in the literature.^8^ In addition, laser polarization has been used (e.g., by Larsen and co-workers^9^) to obtain state-selective photoexcitation of aligned iodine molecules.

Based on this principle, an elegant open-loop control scheme has been demonstrated in which the molecular orientation with respect to the laser polarization direction serves as the control knob.^10^ Transient spatial alignment of the molecular axes is induced at well-defined periods determined by the rotational constants of the molecule under investigation.^11,12^ Consequently, the alignment-sensitive nature of ionization from both frontier and inner-shell molecular orbitals^13−15^ is exploited to ensure that the molecule preferentially resides in a group of selected electronic states in the resulting molecular ion. These electronic states are in turn related to specific photochemical reaction pathways. This scheme has been applied to demonstrate channel-selective molecular fragmentation using a near-infrared (NIR) non-ionizing pump pulse of moderate intensity (typically in the range of 10^12^–10^13^ W/cm^2^) to align the molecule, followed by a second, intense probe pulse at the same wavelength with a controlled time delay to induce molecular ionization and subsequent fragmentation. One drawback of this scheme, due to the intense NIR field, is the inevitable contribution of electron–ion rescattering^16^ to the molecular ionization and successive fragmentation process. Another one is the rather complicated and difficult-to-control strong-field ionization step itself.

On the other hand, high-energy photons delivered by free electron lasers (FELs)^17,18^ and/or high-harmonic generation (HHG) table-top sources^19^ allow for selective excitation of the transiently aligned molecules to specific intermediate states via single-photon absorption, thus leading to a cleaner and simpler process.^20,21^ HHG sources have been applied to investigate the ionization of aligned molecules in the past.^22,23^ However, the simultaneous presence of different harmonics complicates the interpretation of the experimental findings, while harmonic filtering significantly decreases the output intensity of the source.^24^ In contrast, the FERMI seeded FEL delivers ultrafast, intense, wavelength-tunable radiation while exhibiting negligible temporal jitter, a very significant advantage for the implementation of an open-loop active control scheme.

In this work, we demonstrate such a control scheme using the acetylene molecule as a benchmark. Acetylene has been theoretically^25−27^ and experimentally^28−32^ studied in the past by means of pump–probe techniques in the visible and NIR wavelength regions, mainly focusing on the structural rearrangements occurring upon multiple photoionization of the molecular precursor. Time-resolved measurements on the photoisomerization of acetylene induced by extreme UV (XUV) and X-ray laser pulses have also been reported.^33−37^ In the present work, single-photon ionization of transiently aligned acetylene followed by fragmentation of the acetylene cation is investigated. By combining both ion-yield and photoelectron measurements, we show that photoelectrons are a key observable. In our case, the absence of rescattering from the measurements allows for a simpler scheme, obviating the need for more demanding data processing and sophisticated interpretation. Also, the high average flux and flux per pulse, combined with high optical frequency, facilitate the rapid recording of electron and ion signals with high signal-to-noise ratio (SNR), making the scheme presented very flexible and adaptable. Thus, it is the use of seeded FEL pulses that simplifies control of the molecular fragmentation, and although this work is focused on acetylene, its expansion to more complex molecules can be envisaged.

Emphasis is placed on exerting control over the fragmentation of the acetylene cation by using transient molecular alignment to favor the hydrogen fragmentation channel (C_2_H_2_^+^ → C_2_H^+^ + H) over the nondissociative ionization channel. The combination of ion-yield measurements and photoelectron spectra illustrates clearly and unequivocally that ejection of a π electron leaves a stable cation in its wake while the hydrogen fragmentation channel exhibits the same transient alignment behavior as the ejected σ photoelectrons, thus allowing one to distinguish between the two different photochemical processes. These observations are confirmed by state-of-the-art theoretical calculations showing that when the molecule is perpendicular or nearly perpendicular to the polarization direction of the ionizing field, electron ejection from the 1π_u_ orbital (which mainly leads to stable C_2_H_2_^+^ cations; see Figure 1) is the dominant ionization process. In contrast, when the molecule is mainly parallel to the polarization direction, ionization from the 3σ_g_ orbital (which significantly contributes to the C_2_H^+^ + H fragmentation channel; see Figure 1) is comparable in magnitude to ionization from the 1π_u_ orbital.

**Figure 1:**
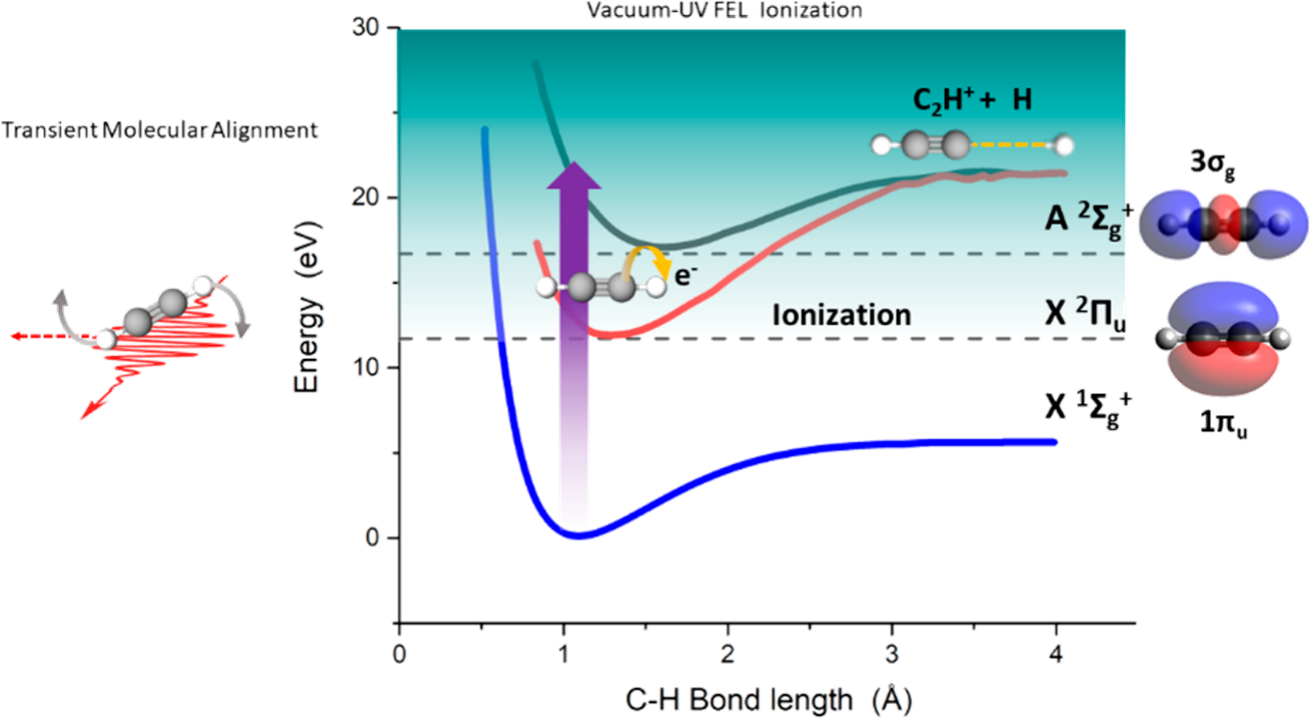
Schematic representation of the open-loop control scheme. The transient molecular alignment is illustrated on the left-hand side of the figure. The potential energy surfaces for the X ^1^Σ_g_^+^ ground state in acetylene and the X ^2^Π_u_ and A ^2^Σ_g_^+^ states in the cation are shown on the right-hand side. The molecular cation remains stable in its ground state upon ejection of a π photoelectron, whereas deprotonation takes place upon release of a σ photoelectron.

The schematic diagram presented in Figure 1 summarizes the adopted open-loop control scheme. The left-hand side illustrates the transient molecular alignment induced by the NIR pump pulse. The right-hand side includes the potential energy curves for the ground state of C_2_H_2_ (X ^1^Σ_g_^+^) and the X ^2^Π_u_ and A ^2^Σ_g_^+^ states (and the corresponding 1π_u_ and 3σ_g_ molecular orbitals, respectively, from which electrons are ejected) of the acetylene cation, in which the molecule is preferentially left after photoionization (the B ^2^Σ_u_^+^ state can also be accessed, but the ionization cross section for this channel is significantly smaller; see Figure 3). The C_2_H_2_^+^ → C_2_H^+^ + H dissociative channel is expected to be associated with the ejection of a photoelectron from the 3σ_g_ molecular orbital, since the potential energy curve of the resulting A ^2^Σ_g_^+^ cationic state has a shallow minimum and its dissociative states can efficiently be reached in a vertical transition from the X ^1^Σ_g_^+^ ground state within the Franck–Condon region. In contrast, the release of a 1π_u_ photoelectron should mainly result in a stable cation in the X ^2^Π_u_ state because its dissociative states cannot be reached via a vertical transition from the ground state.

In the present experimental campaign, an 800 nm (pump) laser field was used to induce impulsive molecular alignment, hence launching a rotational wave packet in the neutral acetylene. By applying a time-delayed FEL (probe) pulse at a wavelength of 53.2 nm (23.29 eV) and scanning over the interpulse time delay (Δτ), the modulations observed in both the ion and photoelectron signals were monitored. At time delays falling in the vicinity of the full revivals τ_r_, half revivals τ_r_/2, and quarter revivals τ_r_/4, strong excursions of the signals are observed. The revival times are related to the rotational constant *B* as τ_r_ = (2*cB*)^−1^, where *c* is the speed of light *in vacuo* and *B* is given in cm^–1^. The ion-yield time of flight (TOF) signal corresponding to the C_2_H_2_^+^ cation, measured upon single-photon ionization of the aligned acetylene molecule, in the vicinity of the half-revival time interval (∼7 ps), is shown as data points in Figure 2a. In order to estimate the degree of alignment, the ⟨cos^2^(θ)⟩ parameter was calculated, where θ denotes the angle between the laser polarization and the molecular axis. This parameter, which was obtained by means of the LIMAO software package,^38^ is shown in the same figure (Figure 2a). The following simulation parameters were used: the rotational constant *B* was 1.18 cm^–1^, the peak alignment laser intensity was 7 × 10^12^ W/cm^2^, the (estimated) rotational temperature was 10 K, and the polarizability orthogonal to the molecular axis was 2.9 Å^3^, with a parallel polarizability of 4.7 Å^3^. The oscillations of the experimental signal are out of phase with the simulation. This behavior has previously been reported in the literature.^39,40^ Specifically, Hasegawa and co-workers^39^ attributed this observation to the shape of the 1π_u_ highest occupied molecular orbital (HOMO) and the fact that, as we will see below, ionization from this orbital is more effective when the laser polarization direction is perpendicular to the molecular axis. Then the ⟨cos^2^(θ)⟩ parameter should be replaced by ⟨sin^2^(θ)⟩ = 1 – ⟨cos^2^(θ)⟩. It should be noted that the authors in refs (39) and (40) used an intense NIR laser field with parameters such that the ionization step resides in the tunneling regime. In our case, although the dominant process is single-photon ionization, the introduction of a π phase factor is still required and has been applied. As can be seen in Figure 2a, the molecular axis is mostly aligned parallel to the polarization of the NIR laser field at a time delay of ∼6.7 ps, corresponding to the valley below 7 ps, whereas the molecular axis is mostly aligned perpendicular to the polarization of the NIR laser field at a time delay of ∼7.6 ps, corresponding to the peak above 7 ps.

**Figure 2:**
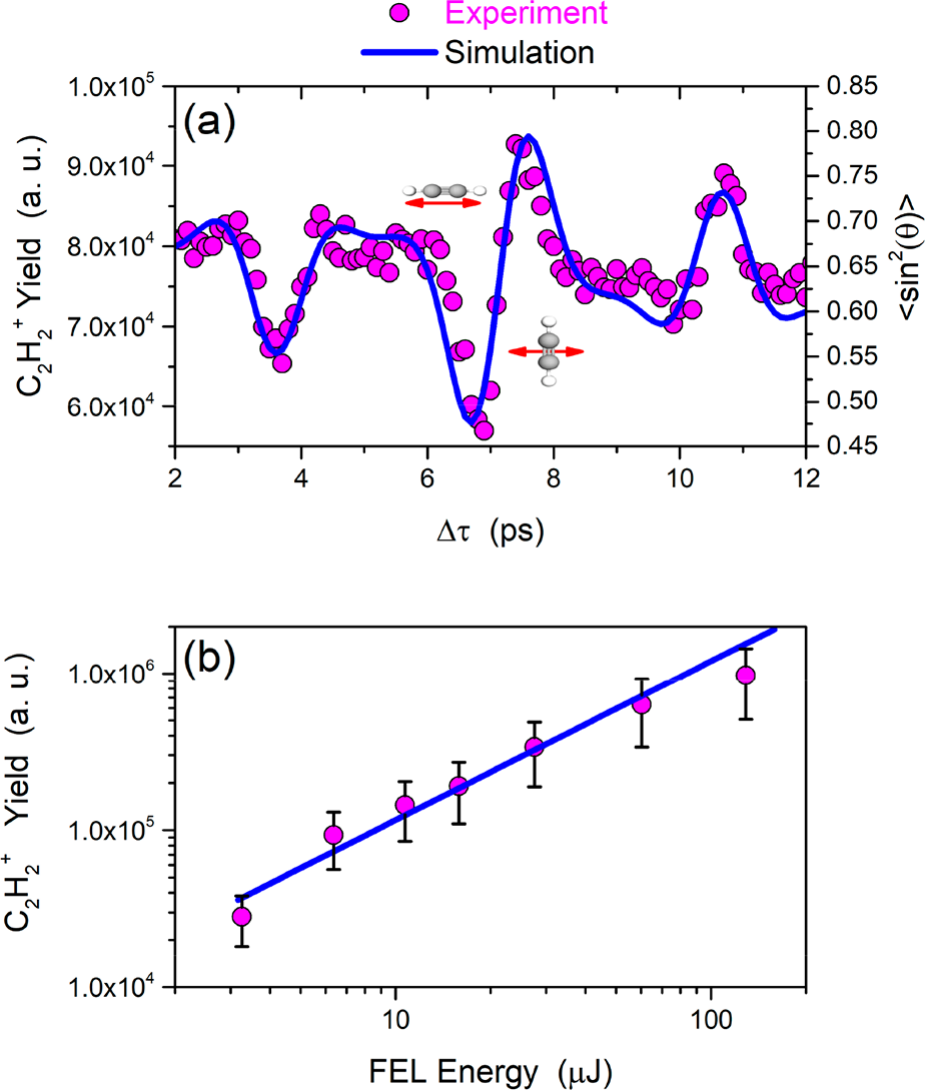
(a) Ion yields (data points) and simulations (solid curve) for different time delays between the 800 nm pump and the VUV FEL probe pulses. (b) Log–log plot of the signal corresponding to the acetylene cation as a function of the FEL pulse energy. A slope of unity (1.02 ± 0.09) is obtained, indicating that a single-photon process accounts for the cation signal.

In order to investigate the nature of the process via which the singly charged molecular ion is produced, the log–log plot of the cation signal for a set of different FEL intensities is presented in Figure 2b. The slope of the linear fit indicates that the acetylene cation is produced by a single-photon process (slope equal to unity). This is critical, as it allows for a rather simplified control scheme, in clear contrast to the case of NIR strong-field ionization. Experimental measurements regarding the single-photon ionization cross sections at the photon energy used in this work (23.29 eV) support the fact that ionization mainly takes place from the 1π_u_ molecular orbital (ionization potential (IP) = 11.3 eV), as it exhibits the highest partial photoionization cross section in the photon energy region of interest.^41^Figure 3 shows the yields for the 1π_u_, 2σ_u_, and 3σ_g_ photoelectron channels that are accessible^42−44^ for the photon energy used in this experimental campaign, displayed as a function of the interlaser time delay. The calculated photoelectron signals are also shown in the same figure. The baselines of the signals correspond to the case where the NIR laser field was absent and are proportional to the single-photon ionization cross sections as shown in Figure 3. Additionally, Figure 4 shows the ion-yield TOF signals for the C_2_H_2_^+^ and C_2_H^+^ ions. In all cases, the TOF signals were again recorded as functions of the interlaser time delays in the presence and absence of the alignment laser field. The modulations of the signals follow the alignment-sensitive ionization of the molecule.

**Figure 3:**
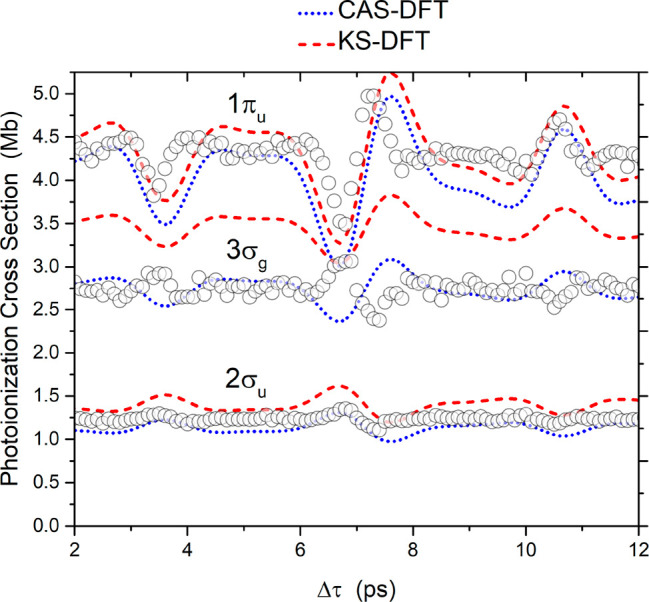
Modulation of the photoelectron signals for all of the open channels (data points) normalized to the various calculated photoelectron cross sections for the following theoretical methods: CAS-DFT (blue dotted line) and KS-DFT (red dashed line).

**Figure 4:**
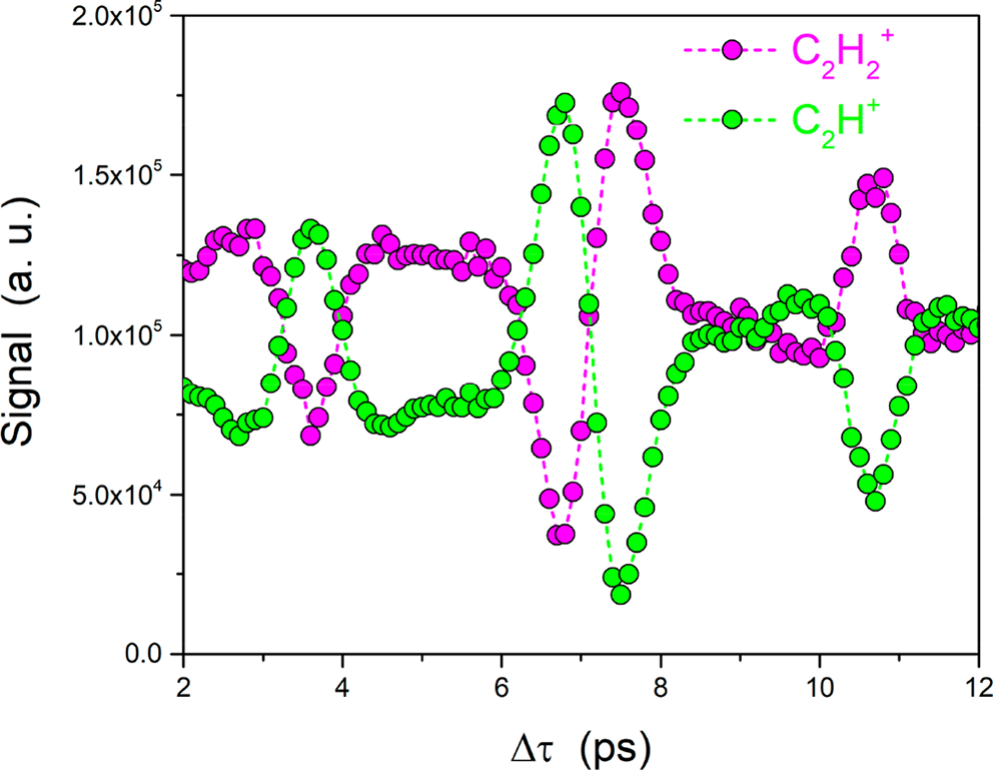
Ion yields for the C_2_H_2_^+^ cation and the C_2_H^+^ fragment for a range of time delays in the vicinity of the half-revival period. Opposite modulation trends are observed for the two fragments. The stable cation follows the same behavior as the π photoelectrons, whereas the C_2_H^+^ fragment tracks the σ photoelectrons.

Figure 3 shows that the photoelectron signal corresponding to emission from the 1π_u_ molecular orbital leading to a C_2_H_2_^+^ cation in the X ^2^Π_u_ state exhibits the strongest modulation. On the other hand, the 3σ_g_ (IP = 16.3 eV) and 2σ_u_ (IP = 18.4 eV) signals, leading to C_2_H_2_^+^ in the A ^2^Σ_g_^+^ and B ^2^Σ_u_^+^ states, respectively, exhibit the same alignment-dependent behavior, which is opposite to that of the π photoelectrons. It can also be observed that while the modulations for the 1π_u_ and 2σ_u_ channels exhibit a high SNR, the observed modulation appears to be somewhat noisier for the 3σ_g_ channel. The photoelectron signals in this figure were obtained in the same way as the ion yields presented in Figure 2, namely, measurements were performed with and without the alignment laser field, and the modulations of the corresponding TOF signals were extracted for every individual channel. Thus, the following process was applied: TOF spectra in the presence of the NIR alignment laser field were compared to spectra recorded in the absence of the alignment field, and the difference signal was recorded as a function of the time delay between the VUV FEL and the NIR field.

With regard to the theoretical calculations, both static-exchange Kohn–Sham density functional theory (KS-DFT) and the more elaborate static-exchange complete-active-space DFT (CAS-DFT) methods were applied to calculate the photoelectron signals shown in Figure 3. In all cases the experimental data were normalized to theory for a direct comparison.

The two methods give rise to the same modulation pattern for each photoelectron channel, although the absolute values of the cross sections are slightly smaller for the CAS-DFT results. The calculated signals are in good agreement with the experimental findings in the case of both the 1π_u_ and 2σ_u_ channels. On the other hand, the calculations do not reproduce the behavior observed for the 3σ_g_ channel. Specifically, there appears to be an opposite modulation pattern between experiment and theory for this channel.

In order to explore this discrepancy further, the partial photoionization cross sections for the 3σ_g_ photoelectrons are presented in Figure SI3 for the two cases where the molecule is aligned parallel (red curve) or perpendicular (black curve) to the polarization direction of the laser field. These results are compared with those obtained using a static-exchange TDDFT methodology (see Experimental and Computational Methods). Previously reported calculations^45,46^ were performed using less sophisticated methods. Although a direct comparison between the older calculations and those presented here is not feasible, one may safely say that the rather large differences among all of these calculations highlight the sensitivity of this measurement in a region of photon energies where the balance of the parallel and perpendicular orientations’ contributions to the 3σ_g_ ionization is critically dependent on the level of theory used. In any case, the fact that both theory and experiment predict that the 3σ_g_ cross section becomes comparable in magnitude to the 1π_u_ one only when the molecules are mainly parallel to the polarization direction of the VUV radiation confirms that orbital-selective, alignment-sensitive ionization has the potential to serve as a control knob. Furthermore, future measurements of the 3σ_g_ signal at different photon energies could help to resolve the disagreement.

The modulation of the ion yields for the C_2_H_2_^+^ cation and the C_2_H^+^ fragment is presented in Figure 4. The signals were obtained using the measured TOF spectra with and without the presence of the alignment laser field. A comparison between the photoelectron signals shown in Figure 3 and the ion yields presented in Figure 4 reveals that the C_2_H_2_^+^ ion signal follows the behavior of the 1π_u_ photoelectrons, meaning that ionization is enhanced when acetylene is aligned perpendicular to the optical laser polarization whereas a lower rate of ionization is observed when the molecular axis is parallel to the laser polarization. Thus, upon removal of a π_u_ electron, the cation is stabilized in its ground electronic state (X ^2^Π_u_). On the other hand, the ion signal associated with the C_2_H^+^ fragment, also shown in Figure 4, exhibits a modulation pattern that follows the 2σ_u_ and 3σ_g_ photoelectron signals and thus is opposite to the modulation observed for the acetylene cation. It is noteworthy that the modulation depth of the two photoion signals is rather pronounced, especially compared to the measurements presented by Xie et al.,^10^ where weaker modulation depths of 10% were observed. For σ photoelectrons, the ionization probability is highest when the laser polarization direction is parallel to the acetylene axis, opposite to the observation for the π photoelectrons. Hence, upon photoionization from the HOMO–1 and/or HOMO–2 σ orbitals, the cation relaxes into one of the two possible excited states (A ^2^Σ_g_^+^ or B ^2^Σ_u_^+^) and follows the dissociation pathway (C_2_H_2_^+^ → C_2_H^+^ + H). This observation highlights the fact that the opposite behaviors of the C_2_H^+^ and C_2_H_2_^+^ fragments with respect to laser alignment allows for control of the molecular fragmentation of the acetylene cation, independent of the actual fragmentation channel. Recent work by Ji and co-workers^47^ on orbital-resolved single ionization of acetylene irradiated by strong NIR laser fields supports the above-mentioned findings: in that case, the authors associated the C_2_H^+^ fragment with the HOMO–1 photoelectron, whereas it is suggested that the stable cation signal is observed as a result of photoionization from the HOMO, leaving the cation in its ground electronic state X ^2^Π_u_. The stability of this ground electronic state of the acetylene cation has been confirmed by theoretical calculations reported in the literature (e.g., the work by Madjet and co-workers^26^), whereas the A ^2^Σ_g_^+^ excited state is associated with acetylene–vinylidene isomerization. Despite the evidence of populating this excited state upon photoionization from the 3σ_g_ molecular orbital, a complete identification of every photochemical process taking place in this experiment is not possible due to the absence of coincidence measurements. Instead, emphasis is placed on providing evidence regarding control of fragmentation of the cation, irrespective of the specific channel. Thus, the C_2_H^+^ and C_2_H_2_^+^ fragments were chosen, as they exhibit the strongest modulation signals.

The potential of such a control scheme on the acetylene cation was suggested by Xie and co-workers,^10^ who mainly focused on the corresponding ion signals for the more complicated case of the dication. The inclusion of photoelectron signals in the present work unequivocally confirms the validity of this control scheme and, as they explicitly indicated, that the alignment-sensitive information associated with the ionization step is imprinted on the ionic fragments.

To conclude, in this work an open-loop control scheme based on transient molecular alignment has been applied to steer the fragmentation of the acetylene cation induced by single-photon absorption of FEL photons in the VUV spectral region. In contrast to previously reported measurements using such a control scheme, where strong-field ionization initiates the fragmentation process, the use of VUV radiation guarantees that no rescattering is induced in this experiment. Importantly, photoelectron measurements were also performed in order to ensure the validity of this control scheme. More specifically, the findings presented in this work suggest that the alignment-sensitive information carried by the ejected photoelectrons is imprinted on the C_2_H^+^ and C_2_H_2_^+^ fragments. The two photoion channels exhibit a pronounced modulation depth, as observed in Figure 4, highlighting that the molecular alignment can be used to control the fragmentation of the precursor. Overall, this work took advantage of the favorable FEL photon properties to demonstrate a simple open-loop control scheme of fragmentation of the acetylene cation.

Importantly, FEL sources can deliver ultrashort pulses carrying high photon fluxes (>10^12^ photons/pulse) right across the XUV/X-ray spectral region from tens to thousands of electronvolts. Wide wavelength tunability, with ideally a narrow bandwidth,^18^ combined with a high photon flux/pulse opens up a plethora of possible applications.^48^ For example, the water window region extending from the carbon K-shell absorption edge (284.2 eV) to the oxygen K-shell absorption edge (543.1 eV) is particularly important for investigation of *in vitro* biological processes. Another possibility concerns the study of biomolecules in the vapor phase. However, working with biomolecules is an inherently challenging task: such molecules typically have very low vapor pressures at temperatures where thermally induced dissociation can be avoided. That limitation noted, the availability of a sufficiently high photon flux allows one to quickly obtain high-quality, low-noise experimental data^49^ on the resulting photoionization/fragmentation processes, which in turn opens up the possibility for extending this control scheme to more complex molecules in the future.

## Experimental and Computational Methods

The measurements were carried out at the Low Density Matter (LDM) end station of the FERMI FEL facility.^50−52^ In order to obtain a cold supersonic molecular beam, the acetylene gas was premixed with He and expanded into the vacuum chamber by means of an Even–Lavie valve at a pressure of 30 bar. The concentration of acetylene in the mix was ca. 2%.

The optical pump laser used to achieve the molecular alignment operated at a repetition rate of 25 Hz and a central wavelength of 800 nm. The pulse duration was ca. 100 fs (FWHM), and the beam was focused down to a diameter of ca. 100 μm (2σ), resulting in a peak intensity of approximately 7 × 10^12^ W/cm^2^. The FEL photon energy was tuned to 23.29 eV, and the FEL operated at a pulse repetition rate of 50 Hz with an average pulse energy of 130 μJ (at the source). The FEL pulse duration was 100 fs (FWHM), and the beam diameter at the focal spot was approximately 50 μm (2σ), resulting in a peak intensity of approximately 1 × 10^12^ W/cm^2^. The photon energy was chosen in order to avoid the adjacent 1s → 3p resonance transition in He at around 23 eV.

Additionally, Sn filters were used to suppress any second-harmonic FEL radiation that might be present in the beam. The ion and electron spectra were acquired using the magnetic-bottle spectrometer installed at the LDM beamline.^53^ The coarse spatial overlap between the pump and probe beams was ensured by use of a retractable fluorescent Ce:YAG screen located in the interaction region. In addition, the coarse temporal overlap (i.e., within 10 to 20 ps) was achieved by means of a retractable antenna, also placed in the interaction region. Fine spatial and temporal alignment was achieved by optimizing the signal due to resonant two-photon, two-color ionization of He. Specifically, the FEL photon energy was tuned at 23.75 eV, resonant with the 1s^2^ → 1s4p transition. Then, upon absorption of an 800 nm photon from the optical laser, He was ionized. Hence, the delay between the two pulses was scanned while monitoring the He^+^ signal.

Photoionization cross sections for a given orientation of the acetylene molecule with respect to the polarization direction were obtained for the three accessible channels (1π_u_, 2σ_u_, and 3σ_g_) in the framework of first-order perturbation theory and within the fixed-nuclei approximation. At the photon energies considered in this work, ionization occurs in tens of attoseconds and therefore is much faster than molecular vibration, dissociation, or rotation, which occur in time intervals ranging from tens of femtoseconds to picoseconds. Therefore, in the calculations, one can safely assume that the nuclear positions remain fixed during the ionization process. In addition, since we are considering ionization from the ground state of the molecule, only molecular geometries close to the equilibrium one, i.e., belonging to the Franck–Condon region, will significantly contribute. For this reason, in all of the theoretical calculations we exclusively considered ionization from the equilibrium geometry (the fixed nuclei approximation). This approximation has been shown to work very well to evaluate total ionization cross sections such as those reported in this work.^54−57^ The corresponding bound–continuum transition matrix elements were evaluated using two different approaches: (i) static-exchange Kohn–Sham density functional theory (KS-DFT)^57^ and (ii) static-exchange complete-active-space density functional theory (CAS-DFT).^58^ In the first method, bound states of the remaining cation are represented as a single Slater determinant built from the KS orbitals of the ground-state neutral molecule that remain occupied after ionization. In the second method, bound states of the remaining cation are obtained from CASSCF calculations. Then the Dyson orbitals, resulting from the overlap between those states and the ground state of the molecule described at the same level of theory, are actually computed, so that correlation between bound electrons is accurately described. In both cases, the continuum states are evaluated using the static-exchange approximation, in which the wave function of the remaining cation is described as above and that of the ejected electron is represented by an eigenvalue lying in the continuum of the KS Hamiltonian built from the DFT ground-state density. We also performed linear-response static-exchange TDDFT calculations for ionization in the 3σ_*g*_ channel, as described in ref (59). In all cases we used the equilibrium geometry of acetylene reported in ref (60). Ground-state KS orbitals were calculated with the ADF package^61^ using a double-ζ polarization plus (DZP) basis set with the LB94 exchange–correlation functional. Dyson orbitals were obtained from a CASSCF (10,10) scheme (Openmolcas QC package^62^) considering a state average of five states for the cationic channels and a single state for the neutral molecule. In both approaches, the KS orbitals were represented by a multicenter expansion in a basis of radial B-spline functions and spherical harmonics. This multicenter expansion consists of (i) a one-center expansion located at the center of mass of the molecule using a radial box of diameter *R*_0_ = 25 au, a radial knot step size *h* = 0.33 au (for a total of 85 B-splines), and spherical harmonics with maximum angular momentum *L*_max_ = 25 and (ii) off-center expansions in each atomic center with a box diameter *r*_0_ = 0.7 au and maximum angular momentum *l*_max_ = 2. Finally, the connection between the different molecular orientations θ and time delays Δτ was made using the experimental ion yields presented in Figure 2a.

## Supporting Information Available

Details regarding the theoretical calculations and comparison of the calculated cross sections for all of the open photoelectron channels (PDF)
