# Supplementary material for: Controlling Fragmentation of the Acetylene Cation in the Vacuum Ultraviolet via Transient Molecular Alignment
Source: J Phys Chem Lett. 2022 Dec 23;14(1):24–31. doi: 10.1021/acs.jpclett.2c03354 (PMC9841558; doi:10.1021/acs.jpclett.2c03354)
Supplement: Supplementary file 1 — jz2c03354_si_001.pdf [file jz2c03354_si_001.pdf]

Supporting Information: Controlling Fragmentation of the Acetylene Cation in the Vacuum-Ultraviolet via Transient Molecular Alignment

L. Varvarezos[a*] , J. Delgado-Guerrero[b,c], M. Di Fraia[d], T. J. Kelly[e], A. Palacios[b,f], C. Callegari[d], A. L. Cavalieri[g,h], R. Coffee[i], M. Danailov[d], P. Decleva[j], A. Demidovich[d], L. DiMauro[k], S. Düsterer[l], L. Giannessi[d], W. Helml[m], M. Ilchen[n,o], R. Kienberger[p], T. Mazza[o], M. Meyer[o], R. Moshammer[q], C. Pedersini[d], O. Plekan[d], K. C. Prince[d,r], A. Simoncig[d], A. Schletter[p], K. Ueda[s], M. Wurzer[p], M. Zangrando[d,t], F. Martín[b,c,u], J. T. Costello[a]

[a]School of Physical Sciences and National Centre for Plasma Science and Technology, Dublin City University, Dublin 9, Ireland.

[b]Departamento de Química, Módulo 13, Universidad Autónoma de Madrid, 28049 Madrid, Spain.

[c]Instituto Madrileño de Estudios Advanzados en Nanociencia, Cantoblanco, 28049 Madrid, Spain.

[d]Elettra-Sincrotrone Trieste S.C.p.A., Basovizza, 34149 Trieste, Italy.

[e]Department of Computer Science and Applied Physics, Atlantic Technological University, T91 T8NW Galway, Ireland.

[f]Institute for Advanced Research in Chemical Sciences, Universidad Autónoma de Madrid, 28049 Madrid, Spain.

[g]Institute of Applied Physics, University of Bern, 3012 Bern, Switzerland.

[h]Paul Scherrer Institute, 5232 Villigen PSI, Switzerland.

[i]Linac Coherent Light Source/SLAC National Accelerator Laboratory, Menlo Park, CA 94025, USA.

[j]Istituto Officina dei Materiali IOM-CNR and Dipartimento di Scienze Chimiche e Farmaceutiche, Università degli Studi di Trieste, 34121 Trieste, Italy.

[k]Department of Physics, The Ohio State University, Columbus, Ohio 43210, USA.

[l]Deutsches Elektronen-Synchrotron (DESY), Notkestrasse 85, D-22607 Hamburg, Germany.

[m]Fakultät Physik, Technische Universität Dortmund, Maria-Goeppert-Mayer-Str. 2, 44227 Dortmund, Germany.

[n]Institut fur Physik und CINSaT, Universitat Kassel,Heinrich-Plett-Str. 40, 34132 Kassel, Germany.

[o]European XFEL, Holzkoppel 4, 22869 Schenefeld, Germany.

[p]Physics Department, Technische Universität München, 85748 Garching, Germany.

[q]Max-Planck Institut für Kernphysik, Saupfercheckweg 1, Heidelberg 69117, Germany.

[r]Department of Chemistry and Biotechnology, Swinburne University of Technology, Melbourne 3122, Australia.

[s]Institute of Multidisciplinary Research for Advanced Materials, Tohoku University, Sendai 980-8577, Japan.

[t]Istituto Officina dei Materiali, Consiglio Nazionale delle Ricerche, 34149 Trieste, Italy.

[u]Condensed Matter Physics Center, Universidad Autónoma de Madrid, 28049 Madrid, Spain.

*(e-mail:lazaros.varvarezos2@mail.dcu.ie).

Static exchange Kohn-Sham density functional theory approach (KS-DFT)

The method has been described in[1]. Here we summarize the main equations. As mentioned in the main text, we work in the fixed nuclei approximation. The ground state of neutral acetylene is written as a single Slater determinant in terms of Kohn-Sham orbitals, $\Psi_0 = \Phi_0 = |\varphi_1, . . . , \varphi_N>$, which are obtained after solving self-consistently the Kohn-Sham eigenvalue equations for N electrons:

$$h_{KS}\varphi_i(\boldsymbol{r}) = \varepsilon_i \varphi_i(\boldsymbol{r}) \qquad i = 1,\ldots\ldots,N \qquad (1)$$

$$h_{KS} = -\frac{1}{2}\nabla^2 + V_{nuc} + V_C + V_{XC} \qquad (2)$$

where V_{nuc} is the nuclear attraction term, V_C the Coulomb potential, and V_{XC} the exchange-correlation potential. We employ the asymptotically correct LB94 exchange-correlation potential[2], since it provides a good description of the molecular potential at large distances, which is essential to describe ionization.

The above equations are solved by using the ADF package[3]. The resulting KS orbitals are then written in terms of a multicenter B-spline basis. This basis comprises a large one-center expansion (OCE) complemented by a set of off-center expansions located at each non-equivalent atomic center. Each expansion consists of a product of B-splines[3] for the radial component, multiplied by symmetry adapted linear combination of real spherical harmonics. The long-range one center expansion in the B-splines basis is written as follows:

$$\chi^0_{nlh\lambda\mu} = \frac{1}{r_0} B_n(r_0)\, X_{lh\lambda\mu}(\theta_0,\varphi_0) \qquad (3)$$

where $B_n(r_0)$ are the B-splines radial functions and $X_{lh\lambda\mu}(\theta_0, \varphi_0) = \sum_m b_{lmh\lambda\mu} Y_{lm}(\theta_0, \varphi_0)$ are symmetry adapted linear combinations of real spherical harmonics $Y_{lm}(\theta_0, \varphi_0)$. The $b_{lmh\lambda\mu}$ coefficients are determined by the symmetry of the molecule. In the same way, the off-center expansions are written in the B-spline basis:

$$\chi^i_{nlh\lambda\mu} = \frac{1}{r_i} B_n(r_i)\, X_{lh\lambda\mu}(\theta_i,\varphi_i) \qquad (4)$$

where i runs over the equivalent nuclei.

Continuum states are obtained by promoting one electron from a bound spin orbital φ_α to a continuum orbital $\varphi_{\varepsilon lh}$, being ε the kinetic energy and l, h the angular momenta quantum numbers. The one-electron continuum wave function is a solution of the single particle equation:

$$h_{KS}\ \varphi_{\varepsilon_k lh}(\boldsymbol{r}) = \varepsilon_k \varphi_{\varepsilon_k lh}(\boldsymbol{r}) \qquad (5)$$

which, by means of the Galerkin approach[4], provides the continuum KS orbitals at the desired photoelectron energy. In the static exchange approximation, the N-electron continuum wave function is written as an antisymmetrized product of the (N-1)-electronic state of the cation and the above one-electron wave function:

$$\Phi^N_{\varepsilon\alpha lm}(\boldsymbol{r}) = A[\Phi^{N-1}_\alpha(\boldsymbol{r_1},\ldots,\boldsymbol{r_{N-1}})\varphi_{\varepsilon_k lh}(\boldsymbol{r_N})] \qquad (6)$$

Once the bound and the continuum wave functions have been evaluated, the dipole transition matrix is obtained in the length gauge:

$$D^i_{\varepsilon\alpha lh} = \langle \Phi^N_{\varepsilon\alpha lm} \,|\, \hat{\mu}_i \,|\, \Phi^N_0 \rangle \qquad i = x,y,z \qquad (7)$$

Static exchange complete active space density functional theory approach (CAS-DFT)

The method has been described in detail in[5]. In this case, the (N-1) electronic state of the cation used in eq. (6) is described at the CAS-SCF level, which provides a much better description of electron correlation. This implies that Dyson orbitals Ψ_α^D must be evaluated. These are given by the overlap between the N-electron ground state wave function of the neutral molecule Φ_0^N, and the remaining (N-1) electron wave function for the α state Φ_α^{N-1}:

$$\Psi_\alpha^D(r_N) = \langle \Phi_\alpha^{N-1} \,|\, \Phi_0^N \rangle = \sqrt{N} \int \Phi_\alpha^{N-1}(\boldsymbol{r_1,\dots,r_{N-1}})\, \Phi_0^N(\boldsymbol{r_1,\dots,r_{N-1},r_N})\, d\boldsymbol{r_1},\dots,d\boldsymbol{r_{N-1}} \quad (8)$$

Combining eqs. (6) and (7) with eq. (8), the dipole transition matrix element is given by:

$$D_{\varepsilon\alpha lh} = \langle \Phi_{\varepsilon\alpha lm}^N \,|\, \hat{\mu} \,|\, \Phi_0^N \rangle = \langle \psi_{\varepsilon lh} \,|\, \hat{\mu} \,|\, \Psi_\alpha^D \rangle + \langle \psi_{\varepsilon lh} \,|\, \eta_\alpha^D \rangle \quad (9)$$

Following common practice, we only retain the first term in the RHS of the equation, which is the direct term that contains all the contributions of dipole transitions from bound to continuum one-electron wave functions.

Photoionization cross sections for an arbitrary molecular orientation

For a given differential molecular solid angle $d\Omega_N = \sin\theta_N d\theta_N d\phi_N$ with respect to the molecular axis, which we assume to coincide with the z axis, the one-photon ionization cross section integrated over electron emission angle is given, to first order of perturbation theory and within the dipole approximation, by[6]

$$\frac{d\sigma_\alpha(\varepsilon)}{d\Omega_N} = \frac{\sigma_\alpha(\varepsilon)}{4\pi}[1 + \beta_N(\varepsilon)P_2(\cos\theta_N)] \quad (10)$$

where

$$\sigma_\alpha(\varepsilon) = \frac{4\pi^2\omega}{3\hbar c}\left(\left|D_{0\to\alpha,\varepsilon}^z\right|^2 + 2\left|D_{0\to\alpha,\varepsilon}^x\right|^2\right) \quad (11)$$

and

$$D_{0\to\alpha,\varepsilon}^k = \sum_{l,h} \left|\left\langle \Phi_{0\to\alpha,\varepsilon,l,h}(\boldsymbol{r}) \,\middle|\, \overrightarrow{\mu_k} \,\middle|\, \Phi_0(\boldsymbol{r}) \right\rangle\right|^2 \quad (12)$$

θ_N is the polar angle defining the orientation of the molecule with respect to the polarization direction, ε the photoelectron energy, α the state of the cation, P_2 the second-order Legendre polynomial, $\Phi_0(\mathbf{r})$ the ground state wave function, $\Phi_{0\to\alpha,\varepsilon,l,h}(\mathbf{r})$ the continuum state wave function with angular momentum l and symmetry h, $\overrightarrow{\mu_z}$ and $\overrightarrow{\mu_x}$ the z and x components of the dipole operator, respectively, and

$$\beta_N(\varepsilon) = \frac{2\left(\left|D_{0\to\alpha,\varepsilon}^z\right|^2 - \left|D_{0\to\alpha,\varepsilon}^x\right|^2\right)}{\left|D_{0\to\alpha,\varepsilon}^z\right|^2 + 2\left|D_{0\to\alpha,\varepsilon}^x\right|^2} \quad (13)$$

Integrating Eq. (10) over the azimuthal angle ϕ_N, which is not determined in the experiment, we obtain

$$\frac{d\sigma_\alpha(\varepsilon)}{\sin\theta_N d\theta_N} = \frac{\sigma_\alpha(\varepsilon)}{2}[1 + \beta_N(\varepsilon)P_2(\cos\theta_N)] \quad (14)$$

Using this expression and the data shown in figure 1 (a) of the manuscript it is possible to retrieve the theoretical photoelectron signals as a function of the time delay between the NIR pulse and the FEL pulse.

Photoionization cross sections for the parallel and perpendicular directions with respect to the FEL field

The partial photoionization cross sections for all the photoelectron channels $1\pi_u$ - Fig. SI1, $2\sigma_u$ - Fig. SI2, $3\sigma_g$ - Fig. SI3 are presented for all three theoretical approaches (CAS-DFT - top row, KS-DFT - middle row, TDDFT - bottom row in every figure). The calculated cross sections are presented both for the case where the molecule is aligned parallel to the polarization direction of the laser field (black curve) and where the molecule is aligned perpendicular to the polarization direction of the laser field (red curve). Cross sections for both parallel and perpendicular orientations are necessary and sufficient to calculate cross sections at any arbitrary orientation according to eq. (11) above.

Importantly, according to Fig. SI3 in the case of the $3\sigma_g$ channel, the crossing between the curves representing the parallel and perpendicular molecular alignment occurs in the vicinity of the photon energy region used in this experiment (represented by the black vertical line), thus highlighting the sensitivity of this measurement. In particular, the TDDFT calculations presented in Fig. SI1, suggest that the crossing occurs very close to the selected photon energy. On the other hand, the KS-DFT static exchange and the CAS-DFT static exchange calculations predict that this crossing occurs at lower photon energies (below 20 eV) along with a modulation that is opposite to that observed in the experiment. In contrast, agreement between theory and experiment is observed for the other two channels.

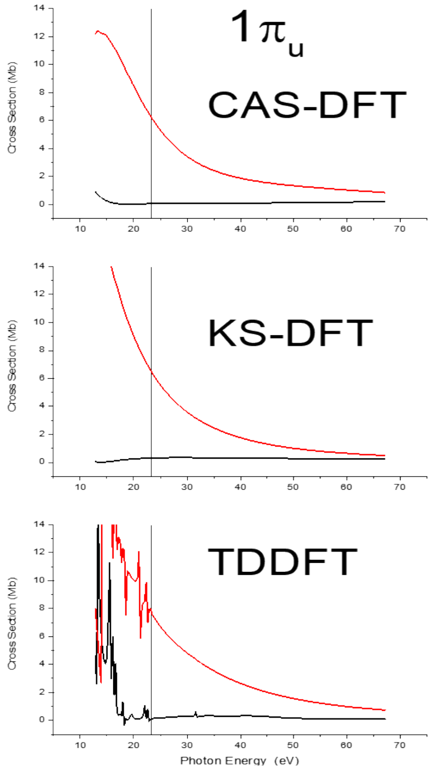

Figure SI1 *Calculated cross sections for the $1\pi_u$ photoelectron channel, and for all the different theoretical methods, CAS-DFT - top row, KS-DFT - middle row, TDDFT – bottom.*

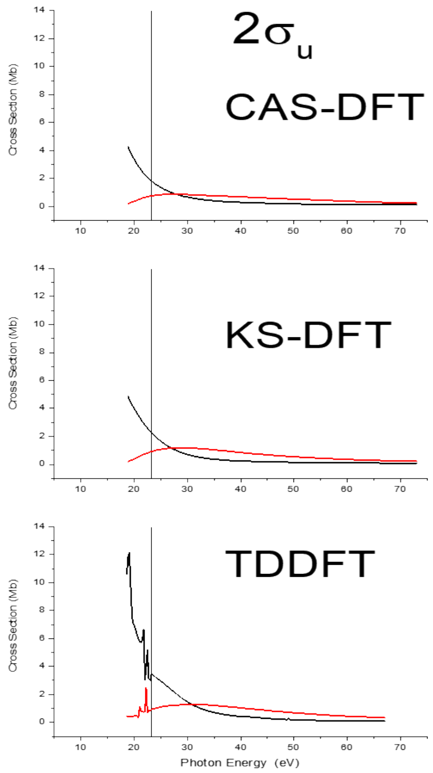

Figure SI2 *Calculated cross sections for the 2σ_u photoelectron channel, and for all the different theoretical methods, CAS-DFT - top row, KS-DFT - middle row, TDDFT – bottom.*

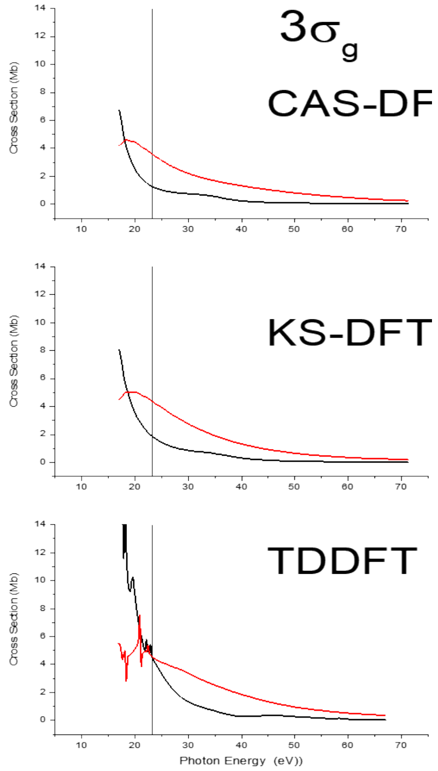

Figure SI3 *Calculated cross sections for the 3σ_g photoelectron channel, and for all the different theoretical methods, CAS-DFT - top row, KS-DFT - middle row, TDDFT – bottom.*

References

(1) Bachau, H.; Cormier, H.E.; Decleva, P.; Hansen, J. E.; F. Martin, F. Applications of B-splines in atomic and molecular physics. *Rep. Prog. Phys.* **2001**, 64, 1815.

(2) Van Leeuwen, R.; Baerends, E. J. Exchange-correlation potential with correct asymptotic behavior *Phys. Rev. A* **1994**, 49**,** 2421 (1994).

(3) ADF2014, SCM, Theoretical Chemistry, Vrije Universiteit, Amsterdam, The Netherlands

(4) Arnold, D.N. An Interior Penalty Finite Element Method with Discontinuous Elements. SIAM J. Numer. Anal. **1982**, 19(4), 742-760.

(5) Ponzi, A.; Angeli, C.; Cimiraglia, R.; Coriani, S.; Decleva, P. Dynamical photoionization observables of the CS molecule: The role of electron correlation. *J. Chem.Phys.* **2014**, 140, 204304.

(6) Dill, D. Fixed-molecule photoelectron angular distributions *J. Chem. Phys.* **1976**, 65, 1130.
